# Chronic sleep deprivation and gender-specific risk of depression in adolescents: a prospective population-based study

**DOI:** 10.1186/s12889-018-5656-6

**Published:** 2018-06-11

**Authors:** Annalijn I. Conklin, Christopher A. Yao, Christopher G. Richardson

**Affiliations:** 1 Collaboration for Outcomes Research and Evaluation (CORE), Faculty of Pharmaceutical Sciences, University of British Columbia, 2405 Westbrook Mall, Vancouver, BC V6T 1Z3 Canada; 2 Centre for Health Evaluation and Outcome Sciences (CHÉOS), Providence Health Research Institute, Vancouver, Canada; 3 School of Population and Public Health, University of British Columbia, Vancouver, Canada

**Keywords:** Sleep deprivation, Longitudinal, Depression, Adolescence, Gender

## Abstract

**Background:**

Chronic exposure to sleep deprivation may increase the risk of depression in young people who are particularly vulnerable to changes in sleep and mental health. Sleep deprivation and incident depression may also differ by gender. We investigated the prospective association between cumulative sleep deprivation and subsequent levels of depressive symptomatology among adolescents from a gender perspective.

**Methods:**

A longitudinal study of 3071 young people in the British Columbia Adolescent Substance Use Survey (BASUS) cohort with three sleep time and two depression measures over 12 months (2011–12). Multivariable linear regression models with interaction terms estimated gender-specific associations between self-reported chronic sleep deprivation and changes in depressive symptomatology; post-estimation analysis calculated adjusted mean depression scores for each level of cumulative sleep deprivation.

**Results:**

Cumulative sleep deprivation was associated with a monotonic increase in depression scores at follow-up in young women, but no consistent pattern was seen in young men. During follow-up, 15% of young women were chronically sleep deprived and 29% were depressed (CESD ≥24). Young women reporting chronic exposure to sleep deprivation had higher CESD scores at follow-up (21.50 points, [CI95 19.55–23.45]), than those reporting no history (16.59 [15.72–17.45]); that remained after multivariable adjustment (19.48 [17.59–21.38]).

**Conclusion:**

Results suggested that chronic sleep deprivation increases the risk of major depression among young women. Mental health promotion for young people should include relevant strategies to ensure young women can achieve recommended amounts of sleep.

**Electronic supplementary material:**

The online version of this article (10.1186/s12889-018-5656-6) contains supplementary material, which is available to authorized users.

## Background

The transition into adolescence has been identified as a period of susceptibility to mental health issues such as depression that can have profound short- and long-term impacts on overall health and well-being. Depression in young people is common, more prevalent among young women and rising in some countries; approximately 5–13% of young males and 10–17% of young females report depressed mood or a major depressive episode in recent years [1–5]. Depressive symptomology typically begins to emerge during mid- to late pubertal stages, particularly for young females [6–9]. The development of depression among youth may lead to substance (mis)use, decreased quality of life, and suicide [6, 10–12]. In addition, the experience of depressive symptomology and disorders during adolescence can increase the risk of more severe outcomes in adulthood including mental disorders and major depressive episodes, unemployment, and attempted suicide [6, 8–10].

Incident depression among young people is attributed to a dynamic and complex interplay of biological, lifestyle, and environmental factors [10]. A key factor that may drive youth depression is changes to sleep patterns and behaviours. During this critical developmental period, multiple neurological and hormonal changes lead to: substantial reductions to slow wave and rapid eye movement sleep; increases in sleep disturbance; a circadian shift to an evening chronotype; and a shortening of total duration of sleep [13]. These neuro-endocrine changes frequently result in young people not obtaining adequate total sleep, referred to as sleep deprivation, which has been linked to important adverse health consequences such as mood disturbances, poor academic performance, increased food intake and weight gain, and engaging in substance use behaviour [14–17].

The comorbidity of depression and of inadequate amounts of sleep is well-established. Much of this evidence on the sleep-depression association is comprised of cross-sectional studies, and little research has investigated the direction of this relationship [13, 18]. Growing epidemiological evidence from prospective studies indicates that short sleep duration precedes depression [19–22]. For instance, a large prospective study of 3134 young people aged 11 to 17 years found that sleep deprivation on weeknights (defined as ≤6 h / night) is associated with almost 40% higher likelihood of reporting symptoms of depression a year later (OR 1.38 [1.02–1.85]), independent of age, gender family, income, and baseline depression [21]. Moreover, when the reverse association was examined, depression did not appear to predict sleep deprivation at follow-up [21]. These findings underscore the potential of addressing sleep duration as a modifiable risk factor for incident depression among young people who are particularly vulnerable to changes in sleep and mental health. However, there is a paucity of longitudinal evidence on the complex relationships between sleep and the development of depression in adolescence as multiple reviews highlight [13, 23]. Furthermore, no studies to date examine gender differences in the association between sleep deprivation and depression despite research indicating that women and men are known to differ in the developmental course of depression, and factors influencing depression risk may be distinct for women and men [8].

We used longitudinal data from a cohort of young people in British Columbia (BC) to examine chronic exposure to sleep deprivation in relation to changes in depression using a gender perspective. We hypothesised that chronic sleep deprivation would be positively associated with higher depression levels at follow-up, and that the relationship would be different for young women and young men.

## Methods

### Study population

In brief, the British Columbia Adolescent Substance Use Survey (BASUS) was a prospective cohort study investigating the psychosocial and environmental factors associated with substance use among 3170 students aged 13–18 years. Online surveys were administered biannually from October 2009 to December 2012 for a total of seven rounds of data collection across 86 secondary schools in the Canadian province of BC, as detailed elsewhere [24]. This study used that last three waves of self-reported data on sleep and wake times during weekdays (2011 fall, 2012 spring, 2012 fall), depression (2011 fall and 2012 fall) and socio-demographics (2011 fall). Students provided written informed consent and the cohort study was approved by the University of British Columbia’s Behavioural Research Ethics Board (#H08–02841).

### Measures

#### Depression outcome

We assessed depression at follow-up using the Centre for Epidemiologic Studies Depression Scale (CESD) score calculated at Wave 7 [25]. The CESD score (range 0–60) is a summation of responses to 20 statements, for which participants rate their agreement based on a 4-point Likert scale: rarely or none of the time (< 1 day); some or a little of the time (1–2 days); occasionally or a moderate amount of the time (3–4 days); and most or all of the time (5–7 days). Higher total scores indicate greater depressive symptomology. We characterised our sample by classifying participants as being depressed or not using CESD scores of 24 or higher that are indicative of the likelihood of developing a major depressive episode [26].

#### Chronic sleep deprivation

Total hours of sleep were calculated from self-reported sleep and wake times on weekdays. We determined exposure to sleep deprivation at each of three time-points (waves 5—7) by constructing a binary variable to classify participants as being sleep deprived (< 8 h) or not sleep deprived (≥ 8 h) according to the National Sleep Foundations guidelines for adolescents [27]. The dichotomised variables contributed to a three-level dose variable of cumulative sleep deprivation comprised of a reference group (not exposed at any time-point), occasional sleep deprivation (exposed at only one time-point), and chronic sleep deprivation (exposed at two or more time-points).

#### Confounders

We accounted for multiple known confounders using covariable data self-reported at study baseline (Wave 5, or Wave 4 for anthropometry). Key covariables included: baseline depression assessed using the CESD (continuous), body mass index (continuous), ethnicity (Caucasian (reference), Asian, Aboriginal, or Other (Latino, Black, and Other)), and family income as perceived relative to peers (far above average / quite a bit average (reference), slightly above average, average, slightly below average, quite a bit below average / far below average).

### Statistical analyses

Descriptive statistics summarised the baseline socio-demographic and health characteristics, and depression scores across levels of cumulative sleep deprivation. The a priori strategy for main analyses was to examine gender-specific associations of chronic sleep deprivation in relation to changes in depression. We ran a series of multivariable linear regression models, beginning with a cross-product term for cumulative sleep deprivation and sex (Male/Female) variables (Model A) and conditioning on baseline CESD scores (Model B); baseline body mass index (BMI) (Model C); ethnicity (Model D); and family income (Model E) [20, 22]. Resulting coefficients of linear regressions were then used in post-estimation calculation of gender-specific adjusted means and 95% confidence intervals (CI95) for each exposure level of cumulative sleep deprivation. The final analytic sample was 3071.

Complete case analyses can reduce statistical power and bias standard errors [28], thus we imputed missing data for all study variables based on procedures outlined by Dong and Peng [29]; 10 imputations were computed assuming missing at random and using the monotone pattern of missing values in SPSS version 24 software (IBM, Chicago IL) [29–31]. We used Little’s MCAR test to confirm that the pattern of missing values was non-systematic (*p* = 0.159) [32]. Our imputation model included baseline and follow-up CESD scores, sleep duration, sleep deprivation, sex, ethnicity, household income, baseline BMI, mother’s education, pubertal stage, and self-rated health; in addition, we incorporated several auxiliary variables (sugary and caffeinated drinks, internet use, leisure-time physical activity, and age) which is recommended to reduce bias and increase efficiency of the imputation model [29].

Separate sensitivity analyses further conditioned on mother’s education (undergraduate/graduate degree, college/trades, high school, some high school), pubertal stage (post-pubertal, late pubertal, mid-pubertal, early puberty, pre-pubertal) [33], and self-rated health (excellent, very good, good, fair, poor). Main models were also re-analysed using only complete cases (range: 559–971). Descriptive statistics were performed using SPSS and inferential statistics were conducted using the ‘margins’ command in Stata version 13.1 (StataCorp LP, College Station, Tx).

## Results

Overall, our sample was 53% female, averaged 14.8 years of age (SD 0.7) at the time of baseline sleep assessment, most (59%) reported having good or excellent health, and 23% were depressed (CESD ≥24). Over a third of our sample had relatively high socioeconomic status: 40% had mothers with a university degree or higher and 42% reported above average family income (relative to peers). There were 921 (30%) young people in our sample of 3071 who were exposed to sleep deprivation, with 17% reporting occasional and 13% reporting chronic sleep deprivation over the 12-month study period. Greater levels of cumulative sleep deprivation were socially patterned (Table 1). Depression at follow up was more prevalent among participants exposed to chronic sleep deprivation compared to those with no history.

**Table 1:** Descriptive characteristics of young people in the BASUS cohort across levels of cumulative sleep deprivation

	History of sleep deprivation		
	None (*n* = 2141)	Occasional (*n* = 533)	Chronic (*n* = 397)
Baseline (Wave 4^*^ or 5)			
Female	51%	54%	61%
Age (years), mean (SD)	14.8 (0.7)	14.8 (0.7)	14.8 (0.7)
White ethnicity	54%	47%	38%
Post-pubertal stage	9%	7%	12%
High family income	44%	39%	37%
Highest maternal education	41%	37%	37%
Excellent/Good Health	62%	52%	51%
BMI (Kg/m^2^), mean (SD)	21.0 (4.0)	20.9 (3.9)	20.8 (3.8)
Sleep duration (hr), mean (SD)	9.0 (0.8)	8.2 (0.9)	7.3 (0.8)
CESD score, mean (SD)	15.2 (10.5)	17.6 (11.0)	18.4 (11.3)
Mid-point (Wave 6)			
Sleep duration (hr), mean (SD)	8.9 (0.8)	7.9 (0.9)	7.0 (0.7)
Follow-up (Wave 7)			
Sleep duration (hr), mean (SD)	8.9 (0.8)	7.7 (0.9)	7.1 (0.7)
CESD score, mean (SD)	15.0 (10.4)	18.2 (11.0)	19.0 (11.9)
Depressed (CESD≥24)	20%	28%	32%

Data on imputed sample (*n* = 3071). *Data on BMI from Wave 4 only. BMI, body mass index (kg/m^2^); high family income was perceived as far above average/quite a bit above average relative to peers; CESD, Centre for Epidemiologic Studies Depression Scale score; Depressed (CESD≥24)

### Cumulative sleep deprivation and change in depression by gender

Our prospective analyses showed a significant and consistent monotonic increase in the change in CESD scores with cumulative sleep deprivation in young women, and no association in young men (details in Additional file 1: Table S1). As shown in Table 2, the mean CESD score at follow-up in young women experiencing chronic sleep deprivation (21.50 [CI95: 19.55–23.45]) was nearly five points higher than those reporting no history of sleep deprivation (16.59 [15.72–17.45]). After conditioning on known confounders, young women reporting chronic sleep deprivation still had significantly higher mean CESD scores (19.48 [17.59–21.38]) than those with no history of sleep deprivation (16.33 [15.42–17.24]). By contrast, mean CESD scores in young men did not differ across levels of cumulative sleep deprivation (Table 2). Table 2 also shows there were significant gender differences in the relationship between cumulative sleep deprivation and levels of depression at follow-up, with young women having higher adjusted mean CESD scores than young men at any exposure level.

**Table 2:** Gender-specific adjusted mean depression scores associated with cumulative sleep deprivation in the BASUS cohort

History of sleep deprivation	Model A		Model B		Model C		Model D		Model E	
	Mean	CI95	Mean	CI95	Mean	CI95	Mean	CI95	Mean	CI95
Young women										
None	16.59†	15.72–17.45	16.37†	15.51–17.23	16.38†	15.51–17.24	16.37†	15.48–17.26	16.33†	15.42–17.24
Occasional	20.62**†	19.03–22.22	19.05*†	17.48–20.61	19.06*†	17.50–20.61	19.07*†	17.59–20.56	19.02*†	17.57–20.47
Chronic	21.50**†	19.55–23.45	19.45*†	17.66–21.25	19.46*†	17.68–21.25	19.54*†	17.72–21.36	19.48*†	17.59–21.38
Young men										
None	13.26†	11.91–14.61	14.36†	13.00–15.73	14.36†	12.98–15.73	14.33†	12.97–15.69	14.38†	13.05–15.72
Occasional	15.25†	12.63–17.87	15.37†	13.39–17.34	15.36†	13.38–17.35	15.38†	13.37–17.38	15.40†	13.39–17.41
Chronic	15.20†	12.55–17.85	15.23†	12.70–17.76	15.22†	12.70–17.75	15.26†	12.70–17.82	15.32†	12.86–17.78

Gender-specific means (CI95) obtained by multivariable linear regression analysis of imputed sample (*N* = 3071) using an interaction term between sex and sleep deprivation (Model A), sequentially adjusted for baseline CESD scores (Model B), baseline BMI (Model C), ethnicity (Model D) and family income (Model E). * *p* < 0.01; ** *p* < 0.001; † *p*-interaction< 0.01

Sensitivity analyses incorporating other potential confounders did not alter the main results (Additional file 1: Table S2). Our complete case analysis also showed chronic sleep deprivation was associated with higher depression levels in young women (17.37 [15.04–19.70]) compared to those with no history of sleep deprivation (13.89 [12.81–14.96]), and no association was found in young men (Additional file 1: Table S3).

## Discussion

This longitudinal study found a monotonic association between cumulative exposure to sleep deprivation and increases in depression among young women only, independent of known confounders including ethnicity and family income. Young women experiencing chronic sleep deprivation reported greater change, and higher mean levels at follow-up, of depressive symptomology than young women with no history of sleep deprivation over 12 months. By contrast, young men showed no differential change in depression, or higher mean levels at follow-up, across levels of cumulative sleep deprivation. Results were robust to adjustment for other potential confounders, and to analysis of complete cases. Our findings therefore supported our initial hypotheses that exposure to chronic sleep deprivation was positively associated with depression and also that the associations were gender-specific. The difference of nearly 5 points in the mean CESD scores between young women with chronic sleep deprivation and those without was a medium effect size (Cohen’s D = 0.20) that became small, though still significant, after considering potential confounders (Cohen’s D = 0.13). Nonetheless, this study contributes new evidence on the distribution of chronic sleep deprivation and its association with changes in depression in youth from a gender perspective.

### Relevance to previous work

Although direct comparisons are difficult due to differences in study design and exposure, our novel findings are consistent with the emerging literature indicating a prospective association between shortened sleep duration and depression in similarly aged populations [19–22]. A previous longitudinal study with three years of sleep and depression data used cross-domain latent growth models to show that students with more total sleep time reported fewer depressive symptoms (β = − 0.20, *p* < 0.001) [19]. Another five-year study used three waves of actigraph data and cross-lagged panel models to demonstrate that increased sleep duration predicted reduced depression (β = − 0.29, *p* < 0.01) and other mental health outcomes [20]. Both longitudinal studies measured depression using the Children’s Depressive Inventory in children under 14 years old [19, 20], and sample sizes ranged from 100 [20] to about 2300 [19]. Two other one-year longitudinal studies assessed sleep deprivation (defined as ≤6 h / night) and risk of depression measured by NIMH Diagnostic Interview Schedule for Children Version IV [21, 22]. Using the Teen Health 2000 cohort of 3134 adolescents aged 11–17 years at follow-up, researchers controlled for multiple confounders and reported that sleep deprivation on weeknights and weekends was linked to a 41% higher odds of depressed mood a year later; and, that sleep deprivation on weeknights alone showed a 23% higher odds of depressed mood [22]. In a similar study that adjusted for baseline depression, sleep deprivation on weeknights was more strongly associated with major depression (adjusted OR 3.76 [1.65–8.58]) than with depressive symptoms (1.38 [1.02–1.85]) at one-year follow-up [21]. Roberts and colleagues also found that the direction of association differed for each depression outcome: reduced quantity of sleep increased the risk for major depression which in turn increases the risk for decreased sleep, but sleep deprivation predicted symptoms of depression and not the reverse [21]. Recently, a very small Australian prospective study, comprising mostly young men (*n* = 138, 64% male), found that total weeknight sleep time at baseline was not associated with CESD depression scores one year later, [34] which concurs with our null finding for young men and highlights the importance of incorporating a gender perspective in this area of research.

There are several interacting factors that might mediate the relationship between chronic sleep deprivation and greater subsequent depressive symptomatology in young women, ranging from biological development to the environmental context. The transition into adolescence has been identified as a period of increased risk for the development of both depression and altered sleeping patterns [13]. Both neurological and hormonal changes associated with puberty not only decrease the total duration of sleep, but also shift the timing and stage of sleep from day to evening chronotype and from deep to lighter stages [13]. Sleep deprivation alone causes the physiological stress pathway (i.e., hypothalamic-pituitary-adrenal axis) to be hyperactive [35], and females are known to be more reactive to stress than males due to the differential effect of testosterone on the hypothalamic-pituitary-adrenal axis [36]. Simultaneously, puberty is associated with cognitive and emotional changes whereby adolescents become more responsive to socioemotional contexts at a time when neural mechanisms for executive functioning and impulse inhibition have yet to fully develop [37]. This differential brain development could influence young peoples’ experiences of depression symptomology [35–37], with young women typically reporting earlier onset and higher levels of depression than young men [7–9]. Overlaying these developmental changes, there are a number of environmental factors as well as other health behaviours that contribute to the sleep-depression paradigm such as early school start times, extensive use of electronic devices, lack of physical activity (which is higher among young women), caffeine and/or alcohol consumption, among others [23].

### Strengths and limitations

We acknowledge several limitations of this study. Cumulative exposure to sleep deprivation was constructed from self-reported sleep and wake times during weekdays, and thus may be subject to various reporting biases such as social desirability, acquiescence, halo effect, midpoint or extreme response styles. However, misclassification of cumulative sleep deprivation from reporting bias would be non-differential as it was unlikely related to CESD scores and hence would have biased results towards the null. Another source of bias is nonresponse from those in lower socioeconomic positions who may be more likely to experience cumulative sleep deprivation and be depressed. Additional measurement error in the CESD scale might underestimate depressive symptoms in young men since the CESD measure is susceptible to differential item functioning (e.g., “I had crying spells”) which results in higher overall scores for females than males [38]. Furthermore, this cohort comprised young people from the Canadian province of BC which potentially limits generalizability of findings, although similar associations have been observed in young populations in a US setting [19–22, 39, 40]. Although we used multiple measurements of sleep to assess chronic exposure to sleep deprivation prior to the measurement of depression at study endpoint, our measure did not determine the timing of, or transitions in, this novel exposure. Finally, our results may also be subject to residual confounding from other potential factors, such as parity and physiologic stress levels, which were not collected in the BASUS cohort study.

Notwithstanding these limitations, our study has several strengths supporting our findings. These include: a longitudinal design with sufficient interval to assess chronic exposure and change in depressive symptomatology; a large and diverse sample similar in socio-demographic and health characteristics to the general adolescent population in BC [41]; adjustment for multiple confounders including known social determinants of health; and multiple imputation to minimize bias in overall estimates. Our study is novel in three important ways. First, we constructed a chronic exposure variable and defined inadequate total sleep according to age-specific guidelines [27], so as to account for the cumulative nature of sleep deprivation in conjunction with a well-validated and widely used measure of depression. Second, it is the first prospective study of chronic sleep deprivation and depression in a developmentally vulnerable group of young people aged 14–17 years. And third, it contributes a gender perspective which is important because the adverse mental health consequences of inadequate sleep duration are more severe for women compared to men [42].

Our findings confirm the importance of young people obtaining at least 8 of sleep per night as recommended by the American Academy of Sleep Medicine for children aged 13 to 18 years [27]. Moreover, the results reinforce proposed school policies involving later start times to accommodate the changes to sleep patterns during this critical developmental stage [23, 43]. And finally, this study further substantiates sleep as an important modifiable behaviour in future health promotion efforts aimed at preventing depressive symptomology given that adolescent depression predicts the frequency and severity of depression in adulthood [8, 9].

## Conclusion

This prospective study showed a strong, gender-specific link between cumulative sleep deprivation and increased depression in an adolescent population. Chronic sleep deprivation was associated with a greater increase in depressive symptoms among young women only. Future public health efforts aimed at promoting young people’s mental health and well-being should consider the use of relevant strategies to address young women’s occasional and chronic sleep deprivation.

## Additional file

Additional file 1: **Table S1.** Change in depression scores and cumulative sleep deprivation in adolescents by gender. **Table S2.** Sensitivity analyses of cumulative sleep deprivation and mean depression in adolescents by gender. **Table S3.** Complete-case analysis of cumulative sleep deprivation and mean change in depression in adolescents by gender. (DOCX 32 kb)

## Abbreviations

BASUS: British Columbia Adolescent Substance Use Survey
BC: British Columbia
CESD: Centre for Epidemiologic Studies Depression Scale BMI Body mass index
