# Supplementary material for: Chronic sleep deprivation and gender-specific risk of depression in adolescents: a prospective population-based study
Source: BMC Public Health. 2018 Jun 11;18:724. doi: 10.1186/s12889-018-5656-6 (PMC5996474; doi:10.1186/s12889-018-5656-6)
Supplement: Supplementary file 1 — Table S1. Change in depression scores and cumulative sleep deprivation in adolescents by gender. Table S2. Sensitivity analyses of cumulative sleep deprivation and mean depression in adolescents by gender. Table S3. Complete-case analysis of cumulative sleep deprivation and mean change in depression in adolescents by gender. (DOCX 32 kb) [file 12889_2018_5656_MOESM1_ESM.docx]

**Table S1. Change in depression scores and cumulative sleep deprivation in adolescents by gender**

|  | **Model A** | | **Model B** | | **Model C** | | **Model D** | | **Model E** | |
| --- | --- | --- | --- | --- | --- | --- | --- | --- | --- | --- |
| **History of Sleep Deprivation** | β | CI95 | β | CI95 | β | CI95 | β | CI95 | β | CI95 |
|  | **Young Women** | | | | | | | | | |
| None | Ref. |  | Ref. |  | Ref. |  | Ref. |  | Ref. |  |
| Occasional | 4.03** | 2.41-5.66 | 2.68* | 1.05-4.31 | 2.68* | 1.05-4.31 | 2.71* | 1.15-4.26 | 2.69* | 1.18-4.21 |
| Chronic | 4.92** | 2.81-7.02 | 3.08* | 1.08-5.09 | 3.09* | 1.08-5.10 | 3.17* | 1.13-5.22 | 3.15* | 1.10-5.20 |
|  | **Young Men** | | | | | | | | | |
| None | Ref. |  | Ref. |  | Ref. |  | Ref. |  | Ref. |  |
| Occasional | 1.99 | -0.27-4.25 | 1.01 | -0.73-2.74 | 1.01 | -0.72-2.74 | 1.04 | -0.72-2.81 | 1.01 | -0.74-2.77 |
| Chronic | 1.94 | -0.93-4.81 | 0.87 | -1.67-3.41 | 0.87 | -1.65-3.39 | 0.93 | -1.67-3.52 | 0.93 | -1.54-3.41 |

Gender-specific coefficients (CI95) obtained from multivariable linear regression analysis of imputed sample (N=3071) using an interaction term between sex and sleep deprivation (Model A), sequentially adjusted for baseline CESD scores (Model B), baseline BMI (Model C), ethnicity (Model D) and family income (Model E). * p < 0.01; ** p < 0.001.

**Table S2. Sensitivity analyses of cumulative sleep deprivation and mean depression in adolescents by gender**

|  | **Model 1** | | **Model 2** | | **Model 3** | |
| --- | --- | --- | --- | --- | --- | --- |
| **History of Sleep Deprivation** | Mean | CI95 | Mean | CI95 | Mean | CI95 |
|  | **Young Women** | | | | | |
| None | 16.34† | 15.43-17.25 | 16.36† | 15.29-17.42 | 16.26† | 15.16-17.36 |
| Occasional | 18.99*† | 17.53-20.45 | 19.06*‡ | 17.46-20.66 | 18.93*‡ | 17.36-20.50 |
| Chronic | 19.45*† | 17.56-21.34 | 19.49*† | 17.56-21.43 | 19.34*† | 17.36-21.32 |
|  | **Young Men** | | | | | |
| None | 14.41† | 13.08-15.72 | 14.33† | 13.00-15.66 | 14.45† | 13.17-15.73 |
| Occasional | 15.39† | 13.40-17.37 | 15.43‡ | 13.32-17.55 | 15.56‡ | 15.39-17.72 |
| Chronic | 15.24† | 12.74-17.75 | 15.34† | 12.94-17.74 | 15.49† | 13.20-17.78 |

Gender-specific means (CI95) obtained by multivariable linear regression analysis of imputed sample (N=3071) using an interaction term between sex and sleep deprivation and adjusting for baseline CESD, baseline BMI, ethnicity, and family income. Model 1 further adjusted for maternal education; Model 2 further adjusted for pubertal stage; and Model 3 further adjusted for self-rated health. * p<0.01; † p-interaction<0.01; ‡p-interaction<0.05.

**Table S3. Complete-case analysis of cumulative sleep deprivation and mean change in depression in adolescents by gender**

|  | **Model A** | | **Model B** | | **Model C** | | **Model D** | | **Model E** | |
| --- | --- | --- | --- | --- | --- | --- | --- | --- | --- | --- |
| **History of Sleep Deprivation** | Mean | CI95 | Mean | CI95 | Mean | CI95 | Mean | CI95 | Mean | CI95 |
|  | **Young Women** | | | | | | | | | |
| None | 14.46† | 13.38-15.51 | 14.10 | 13.18-15.03 | 13.85 | 12.80-14.91 | 13.85 | 12.79-14.91 | 13.88 | 12.81-14.96 |
| Occasional | 21.55**† | 19.57-23.53 | 20.56**† | 18.55-22.57 | 21.73**† | 19.13-24.33 | 21.81**† | 19.20-24.42 | 21.62**† | 18.92-24.33 |
| Chronic | 22.01**† | 19.90-24.12 | 17.74* | 15.79-19.69 | **17.35*** | 15.16-19.55 | 17.63* | 15.40-19.87 | 17.37* | 15.04-19.70 |
|  | **Young Men** | | | | | | | | | |
| None | 10.72† | 9.38-12.06 | 12.78 | 11.57-13.99 | 12.72 | 11.31-14.13 | 12.59 | 11.17-14.01 | 12.77 | 11.33-14.21 |
| Occasional | 13.10† | 10.37-15.83 | 15.30† | 12.37-18.24 | 15.75† | 12.35-19.14 | 15.83† | 12.43-19.23 | 15.05† | 11.55-18.54 |
| Chronic | 12.39† | 8.93-15.86 | 12.95 | 9.42-16.47 | 12.09 | 7.61-16.56 | 12.20 | 7.72-16.68 | 12.64 | 8.13-17.15 |

Gender-specific means (CI95) obtained by multivariable linear regression analysis of complete cases using an interaction term between sex and sleep deprivation (Model A, n=971), sequentially adjusted for baseline CESD scores (Model B, n=806), baseline BMI (Model C, n=577), ethnicity (Model D, n=576) and family income (Model E, n=559). * p < 0.01; ** p < 0.001; † p-interaction<0.01.
